# Generation and Utilization of a Monoclonal Antibody against Hepatitis B Virus Core Protein for a Comprehensive Interactome Analysis

**DOI:** 10.3390/microorganisms10122381

**Published:** 2022-11-30

**Authors:** Yusuke Nakai, Kei Miyakawa, Yutaro Yamaoka, Yasuyoshi Hatayama, Mayuko Nishi, Hidefumi Suzuki, Hirokazu Kimura, Hidehisa Takahashi, Yayoi Kimura, Akihide Ryo

**Affiliations:** 1 Department of Microbiology, Yokohama City University School of Medicine, Yokohama 236-0004, Japan; 2 Advanced Medical Research Center, Yokohama City University, Yokohama 236-0004, Japan; 3 Life Science Laboratory, Technology and Development Division, Kanto Chemical Co., Inc., Isehara 259-1146, Japan; 4 Department of Molecular Biology, Yokohama City University School of Medicine, Yokohama 236-0004, Japan; 5 Department of Health Science, Gunma Paz University Graduate School, Takasaki 370-0006, Japan

**Keywords:** Hepatitis B virus, monoclonal antibody, antibody-based in situ biotinylation, interactome analysis, hypoxia

## Abstract

Hepatitis B virus (HBV) core antigen (HBc) is a structural protein that forms the viral nucleocapsid and is involved in various steps of the viral replication cycle, but its role in the pathogenesis of HBV infection is still elusive. In this study, we generated a mouse monoclonal antibody (mAb) against HBc and used it in antibody-based in situ biotinylation analysis in order to identify host proteins that interact with HBc. HBc antigen was produced with a wheat germ cell-free protein synthesis system and used to immunize mice. Among the established hybridoma clones, a single clone (mAb #7) was selected and further characterized for its ability in the antibody-based in situ biotinylation analysis to collect host proteins that are in the vicinity of HBc. Using mass spectrometry, we identified 215 HBc-interacting host proteins, three of which bind HBc most significantly under hypoxic conditions. Our results indicate that mAb #7 can be used to systematically identify host proteins that interact with HBc under pathophysiological conditions, and thus may be useful to explore the molecular pathways involved in HBV-induced cytopathogenesis.

## 1. Introduction

Hepatitis B virus (HBV) infection is a major global health concern. Approximately 350 million people worldwide are chronically infected with HBV, and an estimated 2 billion are past or present HBV carriers [1,2]. Despite breakthroughs in antiviral therapies, chronic viral hepatitis is still the major cause of liver fibrosis, cirrhosis, and hepatocellular carcinoma (HCC) [3]. Vaccination programs have been established to prevent HBV infection [4], but these are not sufficient to eradicate HBV [5].

HBV, a member of the *Hepadnaviridae* family of viruses, contains 3.2 kb of partially double-stranded DNA that consist of four duplicate RNAs, leading to the production of seven proteins, specifically viral polymerase (P), envelope proteins (S, M, and L), the regulatory X protein (HBx), HBeAg, and the core protein (HBc). HBc is a structural protein required for capsid formation and consists of two major domains, the N-terminal domain (NTD) and C-terminal domain (CTD), the latter of which contains an arginine-rich domain (ARD). While the NTD is responsible for viral assembly, the CTD is important for core particle stability and pgRNA encapsidation [6]. In addition, HBc is involved in various steps of the HBV life cycle, such as intracellular trafficking [7], reverse transcription [8], and cccDNA synthesis [9]. In addition to its diverse functions in viral replication, HBc has recently been shown to play a role in pathogenesis during HBV infection. Previous studies indicated that HBc plays an oncogenic role in HBV-induced carcinogenesis by modifying cellular apoptosis [10] and metabolic pathways [11], actions that facilitate cancer cell growth and malignant transformation, respectively. However, the molecular mechanisms underlying HBc-induced cytopathogenesis are not yet fully understood, and further investigation is needed. In order to achieve this, it is necessary to comprehensively identify the host proteins that interact with HBc in HBV-infected cells.

Protein-protein interactions (PPIs) are essential elements of cellular signal transduction and protein networks [12,13]. Therefore, the analysis of PPIs involving viral proteins is a powerful tool to elucidate the molecular etiology and pathogenesis of virus infection. Affinity purification mass spectrometry (AP-MS), including immunoprecipitation mass spectrometry, is a representative method of PPI analysis [14]. In AP-MS, a target protein fused with peptide tags is overexpressed in cells and the protein complex is purified to analyze the interacting factors by mass spectrometry [13]. Several studies using the AP-MS approach have identified host proteins that interact with viral proteins [15,16,17]. However, the overexpression of a tag-fused protein may result in background noise, causing non-specific PPIs [12]. In addition, it is difficult to detect relatively weak or transient interactions using this method [12].

Recently, proximity-dependent biotin identification (BioID) has been developed as a new PPI analysis technology in which a target protein fused with a modified biotin ligase (BirA) is introduced into cells to label the interacting and proximate proteins [18]. BioID makes it possible to identify cellular proteins that have relatively weak or transient interactions with the target protein under relevant biological conditions [19]. However, it is difficult to target native viral proteins expressed in infected cells since this requires overexpression of BirA-fused viral proteins. In order to overcome this drawback, Bar et al. recently developed a method of in situ biotinylation based on antibody recognition [20]. The advantage of this method is that endogenous proteins are targeted by specific antibodies, followed by proximal biotinylation via hydrogen peroxide activity that can catalyze biotin-phenol for the biotinylation [20,21]. Although this approach enables PPI analysis under physiological conditions, it requires high-quality antibodies to specifically target viral proteins in formalin-fixed cells or tissues.

Hypoxia is an etiologically important microenvironment in liver fibrosis caused by chronic viral hepatitis, cirrhosis, and HCC [22,23]. The progression of chronic HBV infection to chronic hepatitis and cirrhosis induces liver fibrosis and extreme hypoxic conditions in hepatocytes [24,25]. Previous studies have shown that hypoxia-inducible factors (HIFs) can activate the HBV core promoter and enhance HBc expression, thereby increasing the secretion of viral particles under hypoxia in a model system involving cultured liver cells [26]. This suggests that HBV can adapt to hypoxic environments. However, it is unknown if or how HBc plays a role in disease progression under pathological conditions. Therefore, identifying and characterizing host proteins that interact with HBc during hypoxia may help clarify the role of HBc in virus-induced cytopathogenesis.

In this study, we generated a mouse monoclonal antibody (mAb) against HBc and used it in antibody-based in situ biotinylation to identify host proteins that interact with HBc in hypoxic conditions. Our results demonstrate that this mAb can serve as a powerful tool in PPI analysis targeting HBc, and should be useful for elucidating the molecular basis of HBV infection.

## 2. Materials and Methods

### 2.1. Plasmids

The HBV molecular clones pUC19-Ae_US (genotype A), pUC19-Bj_JPN56 (genotype B), pUC19-C_JPNAT (genotype C), and pUC19-D_IND60 (genotype D) have been described previously [27]. HBc cDNAs were amplified from all molecular clones using appropriate primer pairs and subsequently subcloned into the pcDNA-based N-terminal HA vector (Thermo Fisher Scientific Inc., Waltham, MA, USA).

### 2.2. Construction of Wheat Germ Cell-Free Expression Vector

Complementary DNAs encoding the precore/core protein of HBV (genotype Bj_JPN56) were used with a wheat germ cell-free system in order to generate the expression vector for antigen production. The precore/core protein open reading frame encoding amino acids −29 to 183 were amplified by PCR using the corresponding primer pairs. The amplified fragment was cloned into the vector pEU-E01-His-TEV-MCS (CellFree Sciences Co., Ltd., Yokohama, Japan) with restriction enzymes Xho I and Spe I. Deletion mutants of HBV-HBc were generated using the PrimeSTAR Mutagenesis Basal Kit (TakaraBio, Kusatsu, Japan).

### 2.3. Cell-Free Protein Synthesis and Purification

In vitro wheat germ cell-free protein synthesis was performed as previously described [28,29]. WEPRO7240H wheat extract (CellFree Sciences) was used in the bilayer translation reaction, and synthesized proteins were confirmed by immunoblot analysis. N-terminal His-tagged precore/core protein (His-precore/core) was synthesized using a Proteomist XE robotic protein synthesizer (CellFree Sciences) for mouse immunization. The cell-free translation reaction mixture was separated into soluble and insoluble fractions by centrifugation at 15,000 rpm for 15 min. The insoluble fraction was lysed using 8 M urea and then mixed with Ni-Sepharose High Performance beads (GE Healthcare, Waukesha, WI, USA) in the presence of 20 mM imidazole. The beads were washed three times with washing buffer [8 M urea, 20 mM Na-phosphate, 300 mM NaCl] containing 50 mM imidazole. His-precore/core was then eluted in washing buffer containing 500 mM imidazole. Amicon Ultra centrifugal filters (Millipore, Bedford, MA, USA) were used to concentrate purified His-precore/core by approximately 10- to 20-fold.

### 2.4. Immunization and Generation of mAbs

Immunization of BALB/c mice and generation of hybridomas producing anti-HBc mAbs were carried out as previously described [28]. Briefly, purified His-precore/core was injected into BALB/c mice using keyhole limpet hemocyanin as a carrier protein. Four weeks later, lymphocytes were isolated and fused to myeloma cell. Isotype determination was then performed using the IsoStrip Mouse Monoclonal Antibody Isotyping Kit according to the manufacturer’s protocol (Roche Diagnostics, Basel, Switzerland). Purification of mAbs from hybridoma supernatant was performed using a Spin column based Antibody Purification Kit (Protein G) (Cosmo Bio Co., Ltd., Tokyo, Japan). The protein concentration was calculated using a NanoDrop 2000/2000c spectrophotometer (Thermo Fisher Scientific).

### 2.5. Cell Culture

HepG2 cells (JCRB, #JCRB1054), HepG2.2.15.7 cells [30], HepG2-hNTCP-C4 cells [31] and Hep38.7-Tet cells [32] were maintained on collagen-coated dishes with DMEM/F-12 GlutaMAX (Thermo Fisher Scientific) supplemented with 10% fetal bovine serum, 10 mM HEPES, and 5 μg/mL insulin. For hypoxic cultures, cells were adjusted to 1% O_2_ with the BIONIX-1 hypoxic culture kit (Sugiyamagen, Tokyo, Japan) and incubated for 24 h. Normoxic cells were cultured at 5% CO_2_ and 20% O_2_.

### 2.6. HBV Infection

HBV was derived from the supernatants of HepG2.2.15.7 cells, which stably express the HBV genome. The collected supernatants were filtered through a 0.45-μm filter (Millipore) and subsequently concentrated with a PEG Virus Precipitation Kit (BioVision, Milpitas, CA, USA). HepG2-hNTCP-C4 cells were infected with HBV (10,000 GEq/cell) in the presence of 4% PEG8000. After 16 h, the medium was replaced by medium containing 2% DMSO. Eight days after infection, the cells or cell lysates were harvested.

### 2.7. Epitope Mapping and Specificity of mAb #7 Using AlphaScreen Assay

The AlphaScreen assay was performed using 384-well Proxi Plates (PerkinElmer, Boston, MA, USA). Each biotinylated peptide or DHFR (negative control) was mixed in 15 μL of assay buffer [100 mM Tris-HCl (pH 8.0), 0.1% BSA, 0.01% Tween-20] and incubated at 26 °C for 30 min. Next, 10 μL of a detection mixture containing 0.04 μL protein G-conjugated acceptor beads and 0.04 μL streptavidin-coated donor beads (AlphaScreen IgG detection kit, PerkinElmer) were incubated in reaction buffer at 26 °C for 30 min. Antigen-antibody interactions were analyzed using an Envision microplate reader (PerkinElmer).

### 2.8. Bioinformatic Analysis

In order to analyze the frequency of amino acid variation among HBc proteins derived from HBV genotypes A–J, HBc sequences of 13,893 strains were obtained from the Hepatitis B Virus database [33] and aligned using MAFFT software version 7 [34]. The Shannon entropy score was calculated for each position in the protein alignment as previously described [35]. Kaplan-Meier analysis was carried out with the online Xena platform (http://xena.ucsc.edu/) at the University of California, Santa Cruz (UCSC) [36].

### 2.9. Immunoblot Analysis

Cell lysates were separated by 12.5% or 15% SDS-PAGE and transferred onto a PVDF membrane (Millipore). The membrane was then soaked in Tris-buffered saline (TBS) containing 5% (*w*/*v*) skim milk for 1 h and incubated with mAbs (1:1000 dilution) or anti-His polyclonal antibody (1:1000 dilution; GeneTex, Irvine, CA, USA), anti-HA antibody (1:1000 dilution; MBL, Aichi, Japan), anti-Actin antibody (1:1000 dilution; Santa Cruz Biotechnology, Dallas, TX, USA), or anti-ALDOA antibody (1:1000 dilution; Proteintech Group Inc., IL, USA) in TBS containing 0.5% (*w*/*v*) Triton X-100 (TBST) and 5% (*w*/*v*) skim milk at room temperature (RT) for 2 h. After washing three times with TBST, the membrane was incubated for 1 h in 5% (*w*/*v*) skim milk containing anti-mouse IgG-horseradish peroxidase (HRP) antibody (1:5000 dilution; Santa Cruz Biotechnology) or anti-rabbit IgG-HRP antibody (1:5000 dilution; Santa Cruz Biotechnology) or anti-goat IgG-HRP antibody (1:5000 dilution; Santa Cruz Biotechnology). After washing three times in TBST, the blot was detected with ECL Select (Cytiva, Tokyo, Japan) using LuminoGraph II EM (ATTO Corp., Tokyo, Japan).

### 2.10. Immunofluorescence Analysis

HepG2.2.15.7 cells were grown on collagen-coated glass coverslips. HepG2-hNTCP-C4 cells infected with HBV or mock were grown on 96-well plates. HepG2 cells were grown on collagen-coated glass coverslips for 24 h and the cells were transfected with HA-tagged full-length HBc (HA-HBc) of HBV (genotypes A, B, C, and D). The cells were fixed for 15 min in 4% paraformaldehyde (PFA). Next, the cells were permeabilized with 0.1% Triton X-100 in phosphate-buffered saline (PBS) for 10 min at RT, and blocked with 10% normal goat serum (Thermo Fisher Scientific) for 30 min at RT. Then the cells were incubated with supernatants from individual hybridomas (1:50 dilution) or mAb #7 (1:100 dilution) overnight at 4 °C. After incubation with secondary antibody for 1 h at RT, coverslips were mounted using ProLong Gold Antifade Mountant with 4′,6-diamidino-2-phenylindole (DAPI) (Thermo Fisher Scientific). Immunoreactivity was visualized using an FV1000-D confocal laser scanning microscope (Olympus, Tokyo, Japan).

### 2.11. Immunoprecipitation Analysis

Immunoprecipitation analysis was performed according to the method previously described [29]. In detail, cells were harvested and lysed with radioimmunoprecipitation assay (RIPA) lysis buffer [50 mM Tris-HCl (pH 8.0), 150 mM NaCl, 1 mM EDTA, 1 mM dithiothreitol (DTT)] containing complete protease inhibitor cocktail (Roche Molecular Biochemicals, Indianapolis, IN, USA) on ice for 30 min. Cell lysates were cleared by centrifugation at 15,000 rpm for 10 min and immunoprecipitated with 2 µg of mAb #7 mixed with Protein G Sepharose beads (GE Healthcare, Little Chalfont, UK) at 4 °C for 4 h. Bound proteins were analyzed by immunoblotting using mAb #7.

### 2.12. Immunohistochemistry Analysis

HBV (+) and HBV (−) formalin-fixed paraffin-embedded human liver tissues (TissueArray.Com LLC, Derwood, MD, USA) were deparaffinized and rehydrated prior to antigen retrieval. Antigen retrieval was performed in citrate buffer by autoclaving for 10 min at 108 °C. Endogenous peroxidase was inactivated by incubation with 3% hydrogen peroxide in methanol for 15 min at RT. After blocking with Blocking One Histo (Nacalai Tesque, Inc., Kyoto, Japan) for 10 min at RT, the sections were incubated with mAb #7 (1:300 dilution) in TBST containing 20-fold diluted Blocking One-Hist, then incubated with EnVision Dual Link System-HRP (Agilent Technologies, Inc., Santa Clara, CA, USA) for 40 min at RT. Next, the sections were visualized with 3, 3-diaminobenzidine (DAB) reagent (Agilent Technologies) and counterstained with hematoxylin.

### 2.13. Antibody-Based In Situ Biotinylation

Antibody-based in situ biotinylation with mAb #7 was performed by modifying previously reported methods [21]. Briefly, HepG2 or Hep38.7-Tet cells were fixed in 4% PFA for 10 min at RT. Next, cells were permeabilized in PBS containing 0.5% Triton X-100 for 15 min. After washing two times, blocking was performed for 1 h with Blocking One (Nacalai Tesque). Cells were incubated with mAb #7 (1:500 dilution) overnight at 4 °C. Cells were washed with PBS containing 0.05% Triton X-100, then incubated with anti-mouse IgG-HRP antibody (1:5000 dilution; Santa Cruz Biotechnology) for 45 min at RT. After washing with PBS, cells were incubated with biotinylation buffer (200 μM biotin-phenol and 0.0015% H_2_O_2_ in PBS) for 1 min. After washing three times with PBS, cells were lysed and harvested in 1% SDS RIPA buffer [1% SDS, 150 mM NaCl, 1% Triton X-100, 0.5% sodium deoxycholate (Doc), and 50 mM Tris-HCl (pH 8.0)]. Streptavidin MagneSphere Paramagnetic Particles (Promega, WI, USA) were added to the lysate and incubated for 1 h with rotation. Beads were washed sequentially with 0.5% SDS RIPA buffer [150 mM NaCl, 1% Triton X-100, 0.5% Doc, 0.5% SDS, and 50 mM Tris-HCl (pH 8.0)], 0.5 M NaCl RIPA buffer [0.5 M NaCl, 1% Triton X-100, 0.5% Doc, 0.1% SDS, and 50 mM Tris-HCl (pH 8.0)], and 1.2 M NaCl RIPA buffer [1.2 M NaCl, 1% Triton X-100, 0.5% Doc, 0.1% SDS, and 50 mM Tris-HCl (pH 8.0)], then washed again with 0.5% SDS RIPA buffer. The beads were then washed with PBS three times. Finally, the biotinylated proteins were eluted with 4 M Urea buffer [4 M Urea, 50 mM ammonium bicarbonate (NH_4_HCO_3_)].

### 2.14. Sample Preparation for Proteomic Analysis

In order to confirm biotin labeling by antibody-based in situ biotinylation, part of the eluted sample was used for immunoblotting analysis with Streptavidin HRP Conjugate (1:2000 dilution; TOKYO CHEMICAL INDUSTRY Co., Ltd., Tokyo, Japan). For proteomic analysis, the eluted samples were reduced with 10 mM DTT (FUJIFILM WAKO CHEMICAL, Osaka, Japan) at 37 °C for 30 min and alkylated with 25 mM iodoacetamide (FUJIFILM WAKO CHEMICAL) in the dark for 15 min at RT. The samples were diluted from 4 M to 2 M urea in 50 mM NH_4_HCO_3_ and digested with Trypsin Gold (Promega) overnight at 37 °C. Digested peptides were desalted using a StageTip [37], and then dried using a centrifugal evaporator.

### 2.15. Proteomic Analysis and Data Analysis

LC-MS/MS analysis was performed using a TripleTOF 5600 mass spectrometer (AB-SCIEX, Foster City, CA, USA) coupled with an UltiMate 3000 HPLC system (Thermo Fisher Scientific). Peptides were loaded on a trap column (100 μm × 20 mm, C18, 5 μm, 100 Å, Thermo Fisher Scientific) and were subsequently separated on a Nano HPLC capillary column (75 μm × 120 mm, C18, 3 μm, Nikkyo Technos, Tokyo, Japan) at a flow rate of 300 nL/min. Solvent A was 0.1% formic acid in 2% acetonitrile, while solvent B was 0.1% formic acid in 80% acetonitrile. The peptides were eluted using a gradient beginning with 2% B for 0–5 min, then 2% to 40% B for 5–120 min, followed by 95% B for 10 min, and finally equilibration with 2% B for 20 min. The data were acquired using a survey scan performed in a mass range from 400 to 1,250 *m/z* with a scan time of 250 ms. The top 20 peaks were selected for fragmentation. The accumulation time for MS/MS was set to 100 ms, and product ions were scanned in a mass range from 230 to 1800 *m/z*. A list of peaks of detected peptides was generated by Progenesis QI for Proteomics software version 4.2.0 (Waters Ltd., Newcastle-upon-Tyne, UK). The peak lists were searched against human protein sequences in the UniProtKB/Swiss-Prot database (version January 2020) using the Mascot software version 2.7.0 (Matrix Science, London, UK). The search parameters were as follows: trypsin digestion with two missed cleavages permitted; variable modifications: N-terminal acetylation, methionine oxidation, and cysteine carbamidomethylation; peptide charges of 2+, 3+, 4+; peptide mass tolerance ±0.05 Da; and MS/MS tolerance ±0.1 Da. An overall peptide false discovery rate of 1% was set as the threshold for identification.

### 2.16. Statistical Analysis

Statistical differences between normoxic and hypoxic conditions in the proteome analysis were assessed by two-tailed Student’s *t*-test. The outcomes of Kaplan-Meier analysis were evaluated based on *p*-values obtained by the log rank test. *p*-values < 0.05 were regarded as statistically significant.

## 3. Results

### 3.1. Generation of mAbs Targeting HBc Antigen

**Figure 1:**
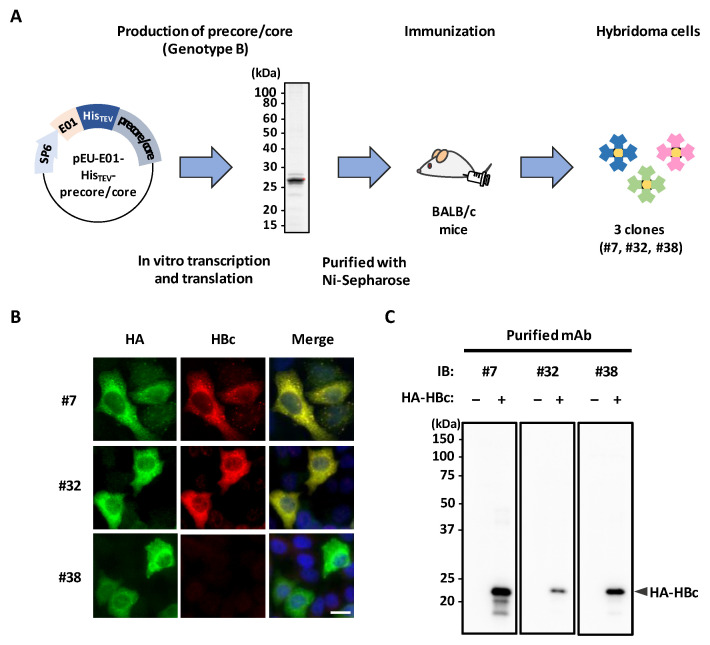
Production and screening of anti-HBc mouse mAbs. (**A**) Schematic of the generation of hybridoma cells that produce anti-HBc mouse mAbs. Recombinant His-precore/core (derived from HBV genotype B) was synthesized by a wheat germ cell-free system and purified with Ni-Sepharose. The red dot indicates the target protein. The purified protein was injected into BALB/c mice. Four weeks later, isolated lymphocytes were fused with myeloma cells, and hybridoma clones were established. SP6, SP6 promoter sequence; E01, translation enhancer sequence; His, histidine-tagged sequence; TEV, TEV protease-recognized sequence. (**B**,**C**) The reactivity of three mAbs (#7, #32, and #38) for HBc. HepG2 cells transfected with HA-HBc were fixed with 4% PFA and stained with the indicated mAbs (hybridoma supernatants, red; HA-antibody, green) and DAPI (blue). Scale bar, 10 μm (**B**). HepG2 cell lysates transfected with HA-HBc were analyzed by immunoblotting with the indicated mAbs (**C**).

To obtain mAbs applicable to antibody-based in situ biotinylation, we first synthesized His-precore/core derived from HBV (genotype Bj_JPN56) using a wheat germ cell-free system (Figure 1A). Purified His-precore/core was confirmed by CBB staining and then used to immunize BALB/c mice. Four weeks after immunization, lymphocytes were isolated and fused with myeloma cells, and finally 48 stable hybridomas were established. Three of these 48 clones (#7, #32, and #38) were selected and further evaluated. In order to evaluate the reactivity for the antigen, we performed immunofluorescence analyses and immunoblot analysis using the three clones. Clones #7 and #32 exhibited specific positivity for the antigen by immunofluorescence analysis (Figure 1B). Since clone #7 (designated here as mAb #7) showed the highest reactivity in immunoblot analysis (Figure 1C), we selected it for further characterization.

### 3.2. Detection of HBc Antigen in HBV Infected Cells

**Figure 2:**
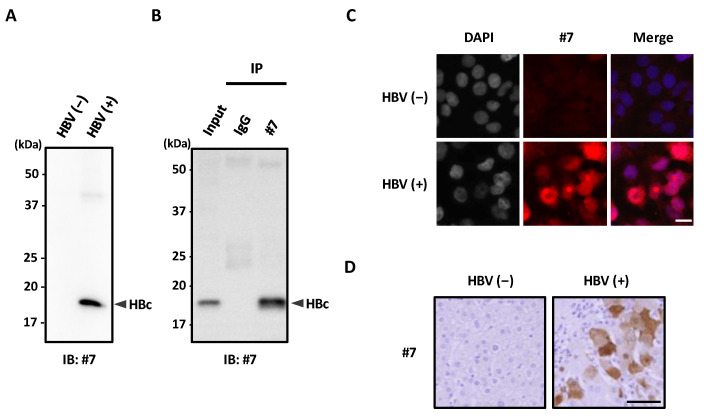
The newly developed mAb #7 detects HBc in HBV-infected cells. (**A**) Lysates of HepG2 cells (HBV (−)) or HepG2.2.15.7 cells (HBV (+)) were analyzed by immunoblotting with mAb #7. (**B**) HepG2.2.15.7 cell lysates were immunoprecipitated with mAb #7, then bound proteins were analyzed by immunoblotting with mAb #7. (**C**) HepG2 cells (HBV (−)) and HepG2.2.15.7 cells (HBV (+)) were fixed with 4% PFA and then stained with mAb #7 (red) and DAPI (blue). Scale bar, 10 μm. (**D**) HBV negative (−) and positive (+) paraffin-embedded human liver tissues were stained with mAb #7 using peroxidase conjugate and DAB chromogen, and then counterstained with hematoxylin. Scale bar, 100 μm.

We next evaluated the effectiveness of our newly developed mAb #7 in several immunoassays. Immunoblot analysis showed that mAb #7 specifically detected HBc derived from HepG2.2.15.7 cells (Figure 2A). We subsequently showed that mAb #7 could be used for immunoprecipitation (Figure 2B). Next, an immunofluorescence analysis demonstrated that mAb #7 clearly detected HBc in HepG2.2.15.7 cells (Figure 2C). We also performed immunoblot [38], immunoprecipitation, and immunofluorescence assays using a HepG2-hNTCP-C4 cell line infected with HBV. Our results showed that mAb #7 detected endogenous HBc in virus infected cells (Supplementary Figure S1A–C). With regard to immunohistochemistry, mAb #7 detected HBc within HBV-infected hepatocytes in paraffin-embedded liver tissue (Figure 2D). In addition, our results revealed that the localization of HBc differed from cell to cell; for instance, some cells exhibited HBc primarily in the nucleus, whereas other cells demonstrated HBc only in the cytoplasm (Supplementary Figure S2A,B). These results confirm that the mAb #7 developed in this study can be useful for analyzing the subcellular localization of HBc. Taken together, these results showed that our newly developed mAb #7 detected endogenous HBc in virus-infected cells and tissues.

### 3.3. mAb #7 Recognizes the Arginine-Rich Domain (ARD) of HBc

**Figure 3:**
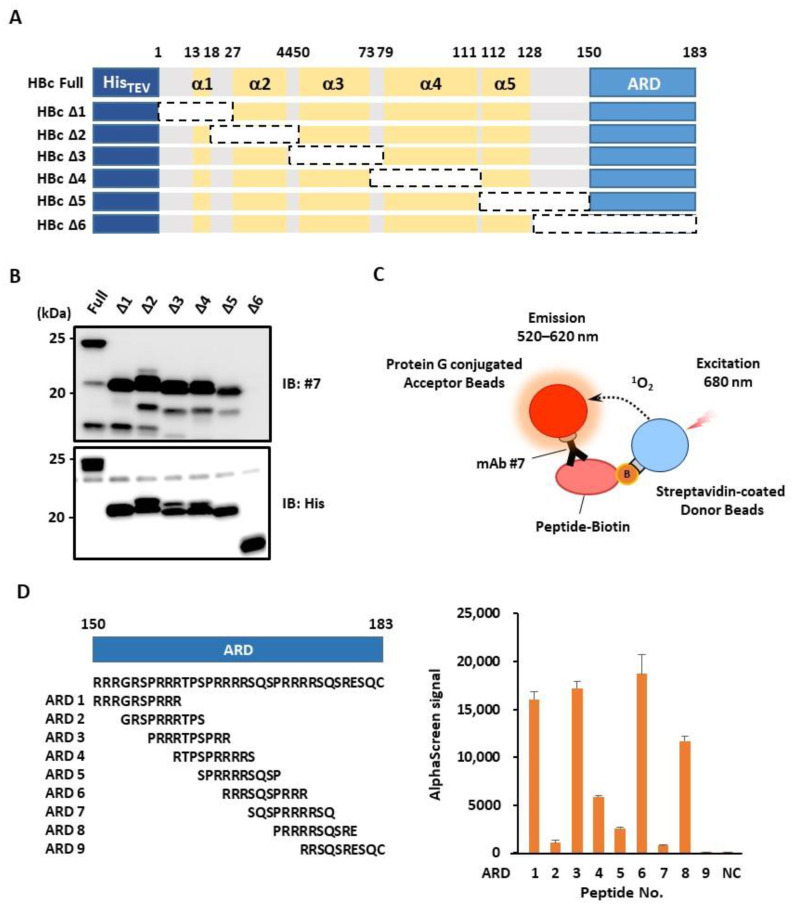
Epitope mapping for the newly developed mAb #7. (**A**) Schematic of a full-length HBc and six deletion mutants (HBcΔ1–6). These N-terminal His-tagged proteins were produced using a wheat germ cell-free system. ARD, arginine-rich domain. (**B**) His-HBc and its deletion mutants were analyzed by immunoblotting using mAb #7 or anti-His antibody. (**C**) Schematic of the AlphaScreen assay. Protein G-conjugated acceptor beads and streptavidin-conjugated donors were used to monitor the interaction of mAb #7 with the synthesized peptides. (**D**) AlphaScreen assay. Nine biotin-tagged ARD peptides (ARD1–9) were synthesized (left panel). The binding activity was measured as the level of the AlphaScreen luminescence signal (right panel). Error bars represent standard deviations from three independent experiments. NC, negative control.

In order to determine the antibody binding site(s) within the antigen, we performed epitope mapping analysis. We first generated six deletion mutants from full-length HBc (HBcΔ1–HBcΔ6) and then performed an immunoblot analysis (Figure 3A). The results revealed that mAb #7 binds to the C-terminal ARD (Figure 3B). To further evaluate the epitope within ARD, we synthesized nine peptides (ARD1–ARD9) and analyzed them with the AlphaScreen assay (Figure 3C). In this case, mAb #7 showed higher signal intensity with four different peptides (ARD1, 3, 6, and 8) (Figure 3D), suggesting that mAb #7 could recognize multiple regions within the ARD of HBc.

### 3.4. mAb #7 Detects HBc Derived from Multiple Genotypes

**Figure 4:**
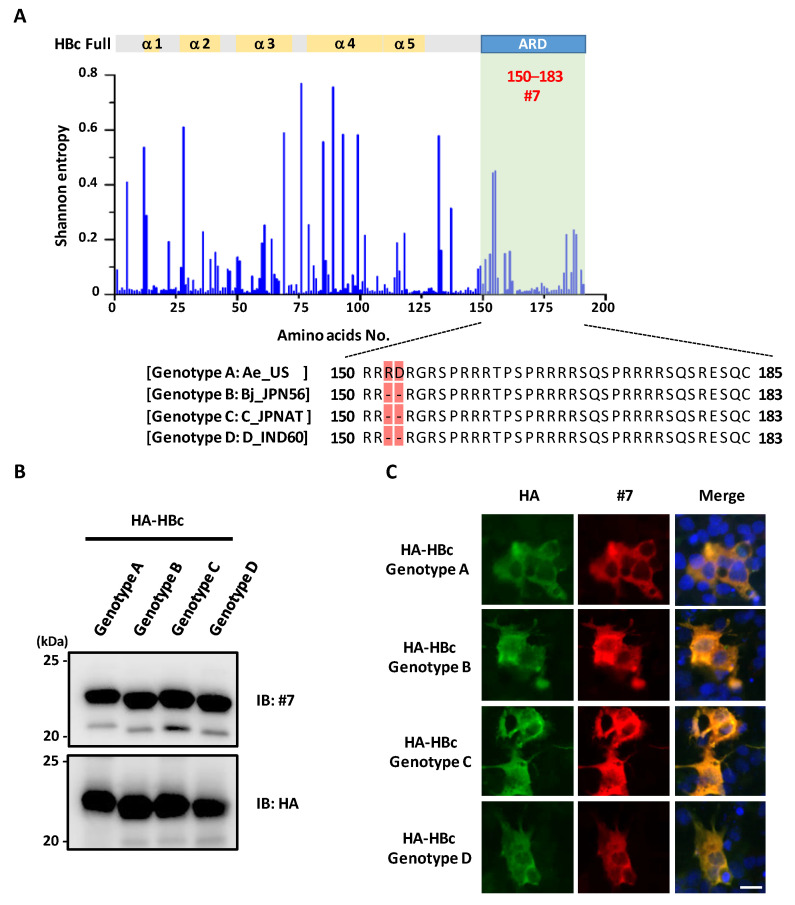
mAb #7 detects HBc derived from multiple HBV genotypes. (**A**) Shannon entropy was calculated for each amino acid residue of HBc derived from 13,893 strains (upper panel). The mAb #7 binding region is represented in green. Multiple alignment of sequences in HBc ARD derived from genotypes Ae_US, Bj_JPN56, C_JPNAT, and D_IND60 (lower panel). The substituted amino acids are highlighted in red. (**B**) Recombinant HA-HBc (HBV genotypes A, B, C, and D) were synthesized using a wheat germ cell-free system and analyzed by immunoblotting with mAb #7 or anti-HA antibody. (**C**) HepG2 cells transfected with HA-HBc (HBV genotypes A, B, C, and D) were fixed with 4% PFA and stained with mAb #7 (red) or anti-HA antibody (green), and then counterstained with DAPI (blue). Scale bar, 10 μm.

We next investigated whether mAb #7 detected HBc derived from multiple HBV strains. Initially, we compared HBc amino acid sequences of 13,893 strains from clinical isolates registered in the Hepatitis B Virus database (Figure 4A). Shannon entropy analysis revealed that the epitope region of mAb #7 was relatively conserved among clinical strains. We next addressed whether mAb #7 could broadly detect HBc from different genotypes (A, B, C, and D). These genotypes have different clinical and virological features; for example, genotype A is known to cause chronic infections more frequently [39,40]. Amino acid alignment showed that the ARD region is similarly conserved among representative strains (Figure 4A). Both immunoblot analysis (Figure 4B) and immunofluorescent analysis (Figure 4C) demonstrated that mAb #7 consistently detected the HBc from all four HBV genotypes.

### 3.5. Antibody-Based In Situ Biotinylation with mAb #7

**Figure 5:**
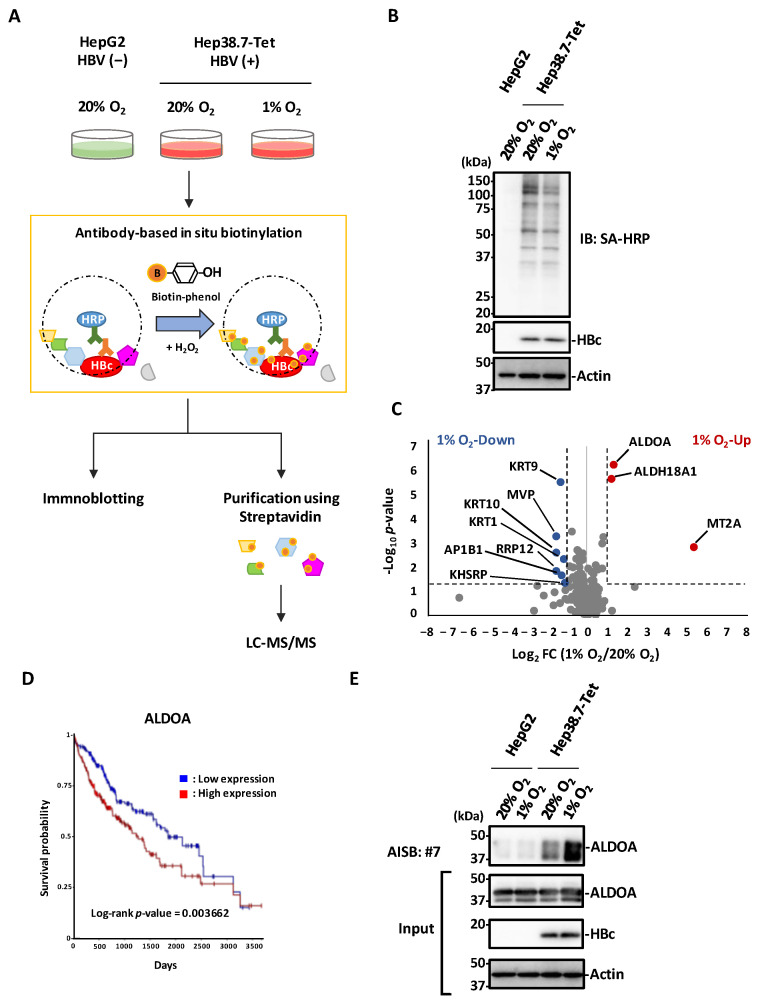
Antibody-based in situ biotinylation analysis with mAb #7. (**A**) Schematic of proteomic analysis for the identification of host proteins that interact with HBc in HBV-positive (Hep38.7-Tet) and HBV-negative (HepG2) cells under normoxic and hypoxic conditions. Each cell was fixed and permeabilized. After mAb #7 bound to HBc, the proximal portion of HBc was biotinylated by adding biotin-phenol and H_2_O_2_. The cell extracts were analyzed by immunoblotting, followed by mass spectrometric analysis of the biotinylated proteins. (**B**) Biotinylation of each cell extract was confirmed by immunoblot analysis with Streptavidin HRP Conjugate. (**C**) Volcano plot representing differences in abundance of 215 host proteins near HBc under normoxic and hypoxic conditions. The *x*-axis indicates the log_2_ fold change and the *y*-axis indicates the –log_10_ *p*-value based on the two-tailed Student’s *t*-test. The proteins that were significantly upregulated (red) or downregulated (blue) under the hypoxic condition were identified based on two criteria: |fold change| > 2 and *p*-value < 0.05. The dotted lines show the criteria. FC, fold change. (**D**) Kaplan-Meier analysis of ALDOA was performed using the UCSC Xena platform and mRNA sequence databases of the Cancer Genome Atlas Program (TCGA). Log-rank *p* = 0.003662. Log-rank *p*-value < 0.05 was regarded as statistically significant. The *x*-axis indicates the days and the *y*-axis indicates the survival probability. (**E**) HBc interacts with ALDOA. The streptavidin-purified biotinylated samples were analyzed by immunoblotting with anti-ALDOA antibody. AISB, antibody-based in situ biotinylation.

Using the newly produced mAb #7, we next performed an antibody-based in situ biotinylation analysis to identify host proteins that interact with HBc under either normoxia or hypoxia (Figure 5A). We first used HBV-expressing (Hep38.7-Tet) or HBV-negative (HepG2) cells to confirm the biotin labeling in the antibody-based in situ biotinylation analysis using mAb #7 (Figure 5B). Multiple bands corresponding to protein biotinylation were detected in HBV-positive cells but not in HBV-negative cells, indicating that only the host proteins proximal to HBc were biotinylated by mAb #7. Interestingly, there was no visually distinct difference in band patterns between normoxic and hypoxic conditions. Subsequently, we purified the biotinylated proteins to identify interacting proteins, and then compared the protein profiles between normoxic and hypoxic conditions. Proteome analysis identified 215 host proteins (Supplementary Table S1), including four proteins (EEF2, KPNB1, NPM1, and FLNB) that have been previously reported to interact with HBc [41]. In this case, 11 proteins showed a significant difference of more than two-fold between the two conditions, and exhibited various intracellular localizations, such as the cytoplasm or nucleus (Supplementary Table S2). Of these, KRT9 and KRT10 were excluded from subsequent analyses because they are known to be common contaminants in sample preparation for MS analysis [42]. Three of the proteins (MT2A, ALDH18A1, and ALDOA) were shown to associate with HBc predominantly under hypoxic conditions, while six (ACLY, MVP, RRP12, AP1B1, KHSRP, and KRT9) associated mainly under normoxic conditions (Figure 5C). We next used the UCSC Xena platform and public mRNA sequence databases to determine whether gene expression of these nine proteins correlated with overall survival of liver cancer patients. Kaplan-Meier analysis showed a significant correlation between survival and mRNA levels for ALDOA (Figure 5D) but not for the eight remaining proteins (Supplementary Figure S3A–H). Therefore, we further investigated the interaction between HBc and ALDOA. Proteins biotinylated using mAb #7 were purified with streptavidin beads. Subsequent immunoblot analysis demonstrated that the interaction between ALDOA and HBc was enhanced under hypoxic conditions (Figure 5E). This indicates that mAb #7 could be useful in comprehensive in teractome analyses of HBc in antibody-based in situ biotinylation.

## 4. Discussion

In addition to its involvement in various viral replication processes, HBc has recently been shown to play a role in the cytopathogenesis of HBV-associated diseases by affecting host signaling pathways [43]. In order to gain a better understanding of the pathogenetic role of HBc in liver diseases, it is important to comprehensively identify host proteins that functionally interact with HBc. In this study, we newly generated mAb #7, a mouse mAb against HBc, and investigated its applicability to antibody-based in situ biotinylation. Our results indicate that mAb #7 can be used with this technique and is useful to explore the molecular pathways relevant to HBV-induced pathogenesis.

Beyond its function as a structural protein, HBc was shown by a number of studies to be involved in various biological processes such as viral replication and pathogenesis [43,44], and to interact with several host proteins [41,45]. While these studies revealed important aspects of HBc function, many employed co-immunoprecipitation of overexpressed tag-fused HBc followed by mass spectrometry [11,46,47], a technique that can cause background noise leading to non-specific PPIs. In order to elucidate the role of HBc in pathogenesis, it is essential to identify host proteins that interact with endogenous HBc under physiological and pathological conditions with proper expression levels and subcellular localization. Antibody-based in situ biotinylation analysis is a recently developed approach that enables proximal biotin labeling using specific antibodies against endogenous proteins [20]. In order to target endogenous proteins, this method utilizes antigen-antibody reactions in fixed cells or tissues for the in situ proximity-dependent biotin labeling [20,21]. In the current study, we produced mAb #7, a high-quality anti-HBc antibody that reacted broadly with the antigens of different HBV genotypes. By targeting HBc antigens that are natively expressed in virus-infected cells, mAb #7 is suitable for antibody-based in situ biotinylation analysis. Indeed, the results of several immunoassays in this study showed that mAb #7 detected endogenous HBc in HBV-infected cells and tissues. Immunohistochemical analysis results also showed that mAb #7 could be useful for detecting subcellular localization of HBc, that occurs predominantly in either the cytoplasm or nucleus. In addition, our results demonstrated that mAb #7 is suitable for antibody-based in situ biotinylation analysis with very slight background noise in the absence of HBV infection.

We demonstrated that mAb #7 recognizes multiple epitopes within the C-terminal ARD. Previous studies reported that the ARD is highly conserved in all genotypes [48], which is consistent with our results. There are 16 arginine residues within the ARD, and 14 of these constitute four distinct subdomains each composed of three to four arginine residues [49]. mAb #7 can react with four oligopeptides, suggesting that it may bind to multiple regions within a single HBc. When used for antibody-based in situ biotinylation, it would be desirable that mAb #7 binds to multiple regions within a single HBc. The association of host proteins with epitope regions of HBc may interfere with antibody binding following in situ biotinylation. If mAb #7 can recognize multiple regions of HBc antigen, this situation will be avoided, making it possible to efficiently biotinylate proximal proteins. However, the data presented here were obtained using synthetic peptides, and further studies are needed to determine whether mAb #7 recognizes multiple or single epitope regions within the ARD of full-length and multimerized HBc.

Hypoxia is one of fibrotic tissue microenvironments that develops in chronic viral hepatitis, liver cirrhosis, and HCC [22]. Several studies have reported an association between hypoxia and HBV. Guidotti et al. found that in HBV transgenic mice, HBc was localized in the nucleus in hepatocytes around the periportal region, whereas around the central vein region with lower oxygen concentrations, HBc was localized not only in the nucleus but also in the cytoplasm [50]. Riedl et al. showed that in liver tissue sections from patients with end-stage chronic hepatitis B, the number of HBc-positive nuclei was higher in hypoxic regions expressing HIF1α than in normoxic regions [25]. These results support the possibility that the functions of HBc differ between hypoxia and normoxia due to its interactions with different host proteins in each condition. Importantly, most previous studies analyzed HBc function in normoxic conditions, which is insufficient to understand the role of HBc in HBV-associated cytopathogenesis. Here, we compared the profiles of host proteins interacting with HBc in normoxic and hypoxic environments. Our antibody-based in situ biotinylation analysis with mAb #7 followed by quantitative proteomic analysis identified 215 host proteins that are proximal to HBc. These included four proteins previously reported to interact with HBc [41], suggesting that antibody-based in situ biotinylation analysis using mAb #7 indeed achieves sufficient biotin labeling of the proteins that are in the vicinity of HBc. We also identified three host proteins (MT2A, ALDH18A1, and ALDOA) that preferentially associate with HBc under hypoxic conditions. MT2A (metallothionein 2A) is a low-molecular-weight (6–7 kDa) protein belonging to a large family of cysteine-rich molecules consisting of 11 functional families [51]. MT2A is known to be induced by hypoxia and to have diverse functions including metal homeostasis, detoxification, oxidative stress protection, and angiogenesis. It was also found to be upregulated in chronic hepatitis and to suppress fibrosis by upregulating collagenase gene expression through activation of stellate cells in liver fibrosis [52]. ALDH18A1 (delta-1-pyrroline-5-carboxylate synthase) is a key enzyme in the proline metabolic pathway. Tang et al. showed that hypoxia significantly increases the expression level of ALDH18A1 in HCC cells in a time-dependent manner, while knockdown of ALDH18A1 significantly induced apoptosis of HCC cells under hypoxia [53]. These results suggest that ALDH18A1 is important for tumor cell survival during hypoxia and for supplying the proline required for the hypoxia response. ALDOA (Fructose-bisphosphate aldolase A) is a key enzyme in the glycolytic pathway that catalyzes the reversible conversion of fructose-1,6-bisphosphate to glyceraldehyde-3-phosphate and dihydroxyacetone phosphate. Zhang et al. reported that aberrant expression of many glycolysis-related genes is associated with the development and recurrence of HCC [54]. These results suggest that HBc may be involved in HBV-related pathogenesis through interactions with multiple host proteins associated with carcinogenesis and liver diseases under hypoxic conditions. However, this study did not fully investigate the functional significance of the HBc-host protein interactions, and therefore further research is needed.

In conclusion, we generated mAb #7, a mouse mAb that targets the HBc antigen and can be used in antibody-based in situ biotinylation analysis. We used mAb #7 to identify host proteins that are potentially involved in HBV-induced pathogenesis. Our data also indicate that the newly produced mAb #7 may serve as an important tool for elucidating the role of HBc in different phases of HBV infection.

## Data Availability

All proteomics data are deposited in the ProteomeXchange Consortium (http://www.proteomexchange.org (accessed on 21 November 2022).) via the jPOST (https://jpostdb.org accessed on 21 November 2022) partner repository (Project ID: PXD036961).

## Supplementary Materials

The following supporting information can be downloaded at: https://www.mdpi.com/article/10.3390/microorganisms10122381/s1. Table S1: List of proteins differentially expressed between normoxic and hypoxic conditions based on quantitative proteome analysis; Table S2: The panel shows host proteins whose abundance differed significantly between normoxic and hypoxic conditions. “Infinity” indicates that only one condition was detected; Figure S1: The newly developed mAb #7 detects HBc in HBV-infected HepG2-hNTCP-C4 cells. (A) The lysates of HepG2-hNTCP-C4 cells infected or uninfected (mock) with HBV were analyzed by immunoblotting with mAb #7. (B) The lysates of HepG2-hNTCP-C4 cells infected with HBV were immunoprecipitated with mAb #7. Bound proteins were subsequently analyzed by immunoblotting with mAb #7. (C) HepG2-hNTCP-C4 cells infected or uninfected (mock) with HBV were fixed with 4% PFA and then stained with mAb #7 (red) and DAPI (blue). Scale bar, 50 μm; Figure S2: The newly developed mAb #7 allows for visualization of the subcellular localization of HBc in HBV-infected liver tissues. HBV-positive paraffin-embedded human liver tissues were stained with mAb #7 using peroxidase conjugate and DAB chromogen, and then counterstained with hematoxylin. mab #7 detects nuclear (A) and cytoplasmic (B) localization of HBc. Scale bar, 10 μm; Figure S3: Kaplan-Meier analysis of eight proteins was performed using the UCSC Xena platform and mRNA sequence databases of the Cancer Genome Atlas Program (TCGA). (A) MT2A, log-rank *p* = 0.4838. (B) ALDH18A1, log-rank *p* = 0.3151. (C) ACLY, log-rank *p* = 0.8405. (D) MVP, log-rank *p* = 0.3204. (E) RRP12, log-rank *p* = 0.05357. (F) AP1B1, log-rank *p* = 0.1882. (G) KHSRP, log-rank *p* = 0.3962. (H) KRT9, log-rank *p* = 0.5700. log-rank *p*-value < 0.05 was regarded as statistically significant. The *x*-axis indicates the days and the *y*-axis indicates the survival probability.
