# Supplementary material for: Generation and Utilization of a Monoclonal Antibody against Hepatitis B Virus Core Protein for a Comprehensive Interactome Analysis
Source: Microorganisms. 2022 Nov 30;10(12):2381. doi: 10.3390/microorganisms10122381 (PMC9783060; doi:10.3390/microorganisms10122381)
Supplement: Supplementary file 1 [file microorganisms-10-02381-s001.zip › 20221130_Supplementary/20221130_Supplementary Figure.pdf]

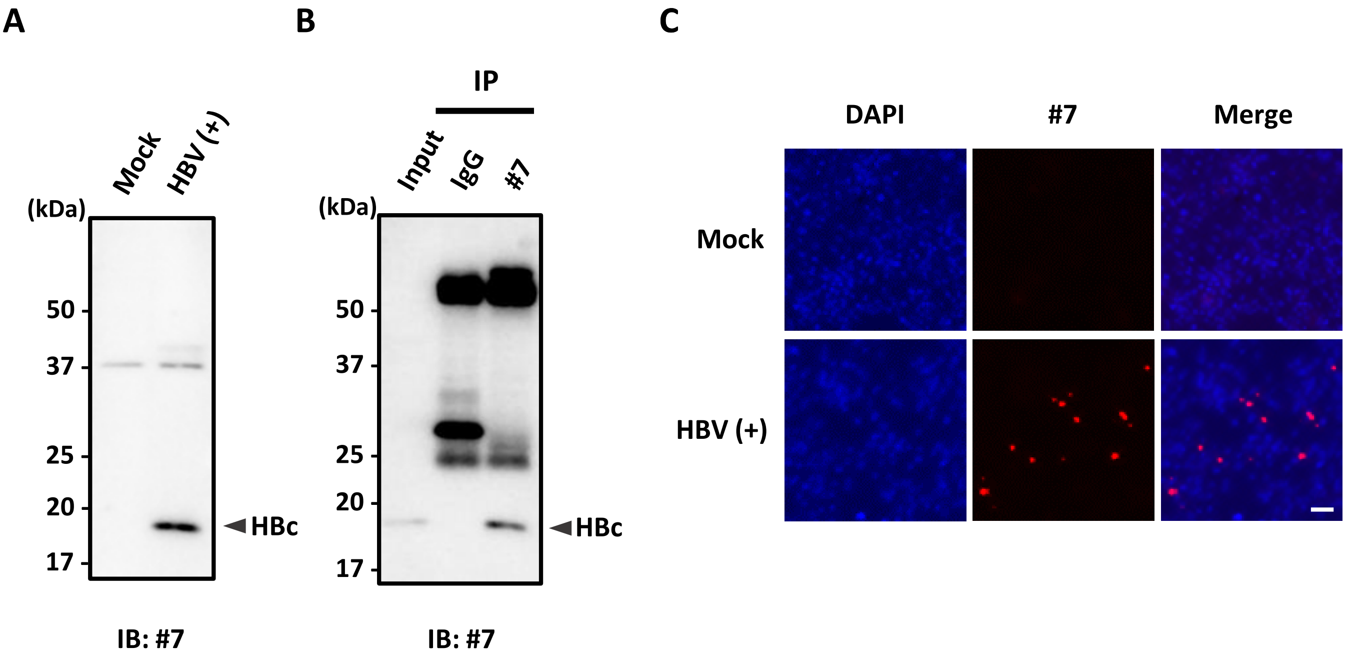

Figure S1. The newly developed mAb #7 detects HBc in HBV-infected HepG2-hNTCP-C4 cells. (A) The lysates of HepG2-hNTCP-C4 cells infected or uninfected (mock) with HBV were analyzed by immunoblotting with mAb #7. (B) The lysates of HepG2-hNTCP-C4 cells infected with HBV were immunoprecipitated with mAb #7. Bound proteins were subsequently analyzed by immunoblotting with mAb #7. (C) HepG2-hNTCP-C4 cells infected or uninfected (mock) with HBV were fixed with 4% PFA and then stained with mAb #7 (red) and DAPI (blue). Scale bar, 50 µm.

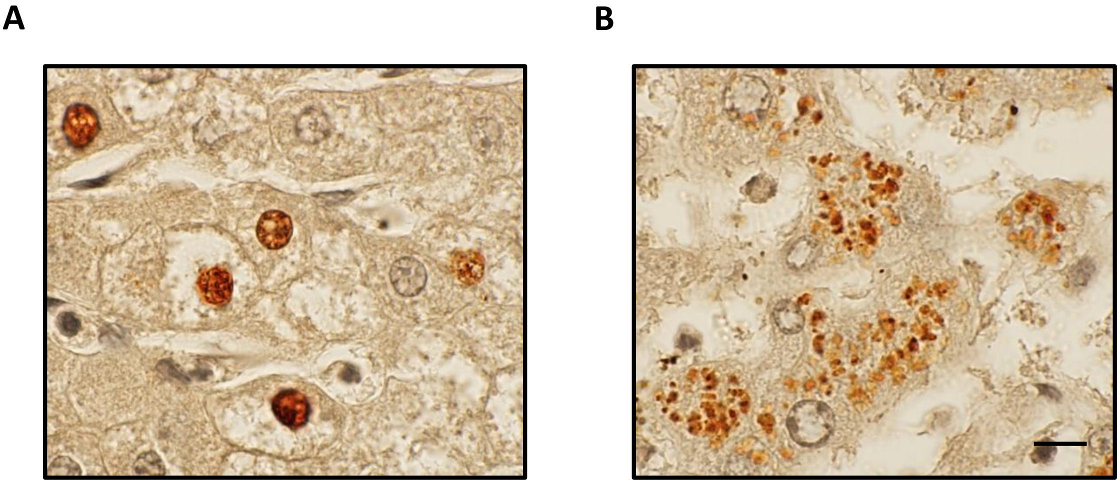

Figure S2. The newly developed mAb #7 allows for visualization of the subcellular localization of HBc in HBV-infected liver tissues. HBV-positive paraffin-embedded human liver tissues were stained with mAb #7 using peroxidase conjugate and DAB chromogen, and then counterstained with hematoxylin. mAb#7 detects nuclear (A) and cytoplasmic (B) localization of HBc. Scale bar, 10 μm.

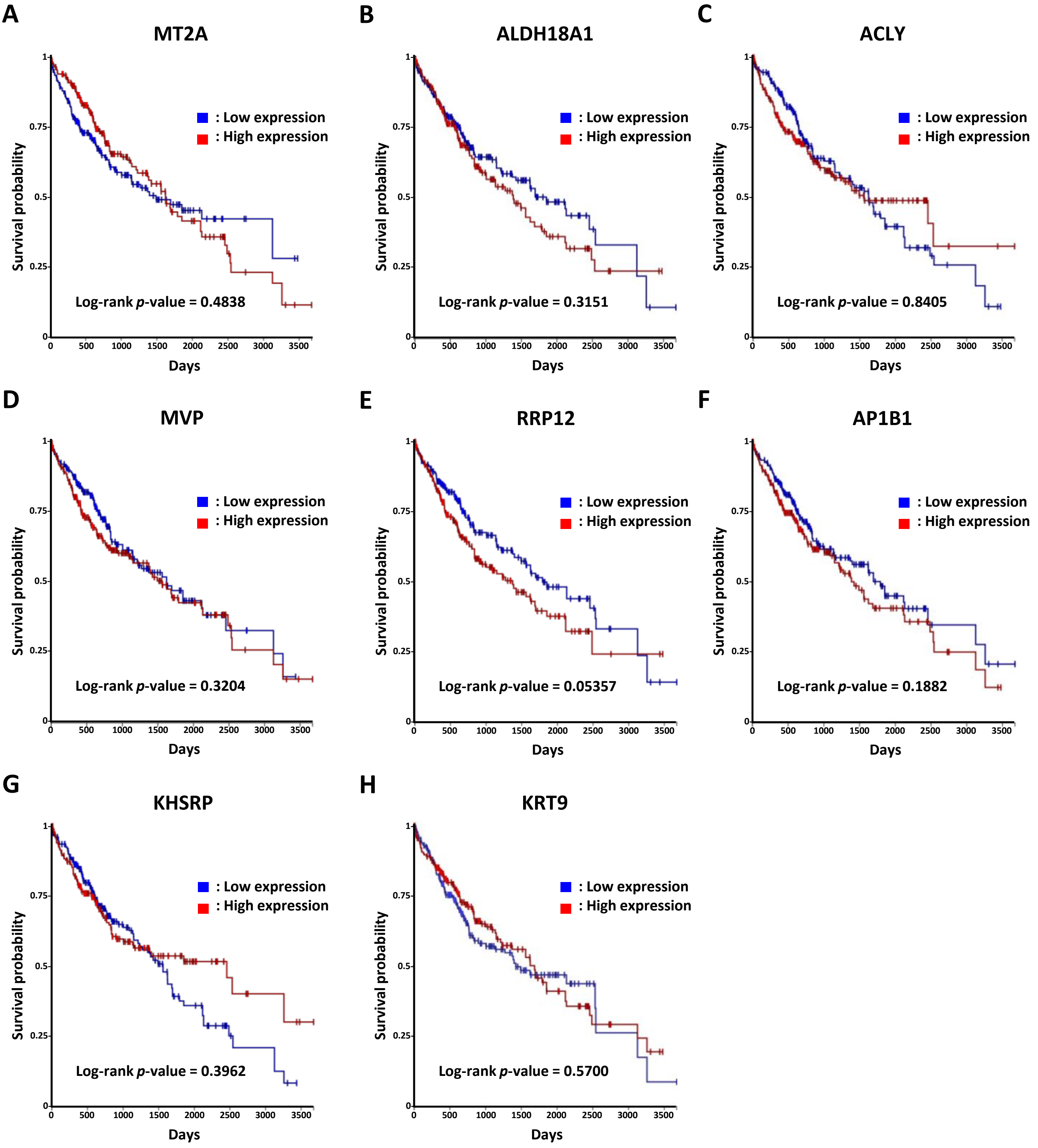

Figure S3. Kaplan-Meier analysis of eight proteins was performed using the UCSC Xena platform and mRNA sequence databases of the Cancer Genome Atlas Program (TCGA). (A) MT2A, log-rank $p = 0.4838$. (B) ALDH18A1, log-rank $p = 0.3151$. (C) ACLY, log-rank $p = 0.8405$. (D) MVP, log-rank $p = 0.3204$. (E) RRP12, log-rank $p = 0.05357$. (F) AP1B1, log-rank $p = 0.1882$. (G) KHSRP, log-rank $p = 0.3962$. (H) KRT9, log-rank $p = 0.5700$. log-rank p-value < 0.05 was regarded as statistically significant. The x-axis indicates the days and the y-axis indicates the survival probability.
